# Geographic variations in avoidable hospitalizations in the elderly, in a health system with universal coverage

**DOI:** 10.1186/1472-6963-8-42

**Published:** 2008-02-18

**Authors:** Purificacion Magan, Angel Otero, Angel Alberquilla, Jose Manuel Ribera

**Affiliations:** 1 Unidad Epidemiología Clínica (CIBERESP; RETICEF), Hospital 12 de Octubre, Madrid, Spain; 2 Catedra de Medicina de Familia UAM/Novartis-Departamento de Medicina Preventiva y Salud Pública (RETICEF), Facultad de Medicina Universidad Autónoma, Madrid, Spain; 3 Departamento de Sistemas de Información Sanitaria, Gerencia Atención Primaria Area 11, Madrid, Spain; 4 Departamento de Gerontología y Geriatría (RETICEF), Facultad de Medicina Universidad Complutense, Madrid, Spain

## Abstract

**Background:**

The study of Hospitalizations for ambulatory care sensitive conditions (ACSH) has been proposed as an indirect measure of access to and receipt of care by older persons at the entryway to the Spanish public health system. The aim of this work is to identify the rates of ACSH in persons 65 years or older living in different small-areas of the Community of Madrid (CM) and to detect possible differences in ACSH.

**Methods:**

Cross-sectional, ecologic study, which covered all 34 health districts of the CM. The study population consisted of all individuals aged 65 years or older residing in the CM between 2001 and 2003, inclusive. Using hospital discharge data, avoidable ACSH were selected from the list of conditions validated for Spain. Age- and sex-adjusted ACSH rates were calculated for the population of each health district and the statistics describing the data variability. Point graphs and maps were designed to represent the ACSH rates in the different health districts.

**Results:**

Of all the hospitalizations, 16.5% (64,409) were ACSH. Globally, the rate was higher among men: 33.15 per 1,000 populations vs. 22.10 in women and these differences were statistically significant (p < 0.05) in each district. For men the range was 70.82 and the coefficient of variation (CV) was 0.47, while for women the range was 43.69 and the CV was 0.48. In 93.1% of cases, the ACSH were caused by hypertensive cardiovascular disease, heart failure or pneumonia. A centripetal pattern can be observed, with lower rates in the districts in the center of the CM. This geographic distribution is maintained after grouping by sex.

**Conclusion:**

A significant variation is demonstrated in "preventable" hospitalizations between the different districts. In all the districts the men present rates significantly higher than women. Important variations in the access are observed the Primary Attention in spite of existing a universal sanitary cover.

## Background

Hospitalizations for ambulatory care sensitive conditions (ACSH) are an indicator of the use of hospital resources for health problems that could have been prevented, treated or controlled in primary health care (PHC) [1]. This indicator was developed in the late 1980s by Billings in the United States to examine access to health care by the indigent population[2]. It is a direct indicator of potentially avoidable hospitalizations and an indirect indicator of access to PHC and its capacity to manage health problems. In fact, several studies have concluded that high rates of ACSH indicate suboptimal PHC, understood as inappropriate care with regard to type, place, intensity or timing of management of the health problem[3,4].

In Spain there is growing interest in evaluating primary care by using health outcome indicators[5] such as ACSH. To understand this interest one must be aware of the profound changes produced in the Spanish health system in the last 25 years, especially with regard to the organization and management of primary care [6]. The Spanish health care system is mainly publicly financed and is designed to provide access to all the country's residents, regardless of their socioeconomic level or geographical differences. It is a highly decentralized system, with 17 Regional Health Services managed by Regional Governments [6]. The primary care level is based in Health Centers where the Primary Health Care Team, made up of doctors, nurses, a social worker and clerical staff, provide care for a population of 5,000–25,000 people (1,700–1,800 persons per family doctor) [6].

Although many small-area studies have been carried out to identify variations in ACSH in persons under 65 [7], few such studies have been made in older persons, despite their importance both demographically and in terms of health resource use[8]. By 2050, Spain will be the country with the largest proportion of population over 60 (44.1%)[9], however no study of ACSH in the older population has been made in this country.

Thus, the study of ACSH has been proposed as an indirect measure of access to and receipt of care by older persons at the entryway to the Spanish public health system. The objective of this work is to identify the rates of ACSH in persons 65 years or older living in different small-areas of the Community of Madrid (CM) and to detect possible differences in ACSH.

## Methods

This was a cross-sectional, ecologic study. Health care in the CM is organized into 11 health areas (with a public reference hospital in each) which are in turn divided into health districts. The health district was used as the territorial unit of analysis. The study covered all 34 health districts of the CM, with a total population of 5,372.433 inhabitants in 2001.

The study population consisted of all individuals aged 65 years or older residing in the CM between 2001 and 2003, inclusive. Three age groups were created for the presentation of results: 65–74 years, 75–84 years and 85 or older.

We analyzed all hospitalizations during the years 2001–2003. The Continuous Municipal Population Census was the source of data for population characteristics (size and structure). Information on hospitalization episodes was obtained from the Minimum Basic Data Set on Hospital Discharges and Ambulatory Surgery (CMBD-AH, in Spanish). The CMBD-AH is a registry of all hospitalization episodes, which includes administrative data (age, sex, residence, type of admission, etc.) and clinical data (principal diagnosis and other secondary diagnoses, diagnostic and therapeutic procedures); all public and private hospitals in the CM are required to report these data. This study was based on hospitalizations in public hospitals, which represent 71% of all hospitalizations in the CM. These data files were supplied by the Health Information Service of the General Directorate of Informatics, Communications and Technological Innovation of the Regional Health Ministry of the Community of Madrid.

Diagnoses and procedures in the CMBD-AH are coded in accordance with the International Classification of Diseases, 9^th ^revision, Clinical Modification (ICD 9-CM). Avoidable ACSH were selected from the list of conditions validated for Spain by Caminal, et al (Table 1) [10].

**Table 1:** Categories of Ambulatory Care Sensitive Conditions (ACSC) used to evaluate the capacity of Primary Care to avoid hospitalizations

**Vaccine-preventable diseases and other diseases**	**ICD-9 Codes**
1. Diphtheria	32
2. Tetanus	37
3. Acute poliomyelitis	45
4. Homophiles meningitis	320.0
5. Rheumatic fever	390; 391
**Syphilis**	

6. Congenital syphilis	90
**Tuberculosis**	

7. Other tuberculosis	012–018
**Diabetes**	

8. Diabetes with general complications	250.1; 250.2; 250.3
9. Hypoglycemic coma	251.0
10. Gangrene+ diabetes with peripheral circulatory disorders	785.4 + 250.7
**Disorders of fluid, electrolyte, and acid-base balance**	

11. Volume depletion/dehydration	276.5
12. Hypotassemia	276.8
**Acute respiratory infections**	

13. Peritonsillar abscess	475
**Hypertensive cardiovascular disease**	

14. Malignant essential hypertension	401.0
15. Malignant hypertensive kidney disease	403.0
16. Malignant hypertensive heart and kidney disease	404.0
17. Malignant secondary hypertension	405.0
18. Ischemic heart disease	410 – 414
19. Cerebrovascular disease	430; 431; 436; 437.2
**Congestive heart failure (CHF)**	

20. Malignant hypertensive heart disease with CHF	402.01
21. Benign hypertensive heart disease with CHF	402.11
22. Hypertensive heart disease, unspecified, with CHF	402.91
23. Heart failure	428
24. Acute pulmonary edema, unspecified	415.4
**Pneumonia**	

25. Pneumonia due to Hemophilus influenza	482.2
26. Pneumonia due to Streptococcus	482.3
27. Pneumonia due to other specified organism	483
28. Bronchopneumonia/Pneumonia, organism unspecified	485; 486
**Bleeding or perforated ulcer**	

29. Acute or chronic gastric ulcer or unspecified.	531.0; 531.2; 531.4; 531.6
30. Acute or chronic duodenal ulcer or unspecified	532.0; 532.2; 532:4; 532.6
31. Peptic ulcer, site unspecified, acute or chronic or unspecified	533.0; 533.2; 533.4; 533.6
**Acute appendicitis with complications**	

32. Acute appendicitis with generalized peritonitis	540.0
33. Acute appendicitis with peritoneal abscess	540.1
**Urinary tract infections**	

34. Acute pyelonephritis	590.1
**Pelvic inflammatory disease**	

35. Inflammatory disease of ovary, fallopian tube, pelvic cellular tissue and peritoneum	614

Source: Caminal et al [10]

Age- and sex-adjusted ACSH rates were calculated for the population of each health district. The population was stratified into 5-year age groups from 65 to 99 years, with a single population group for those 100 years of age or older. The rates were standardized by the direct method, with the 2001 population of the CM used as the reference population. The data for the three years of the study were combined to produce more stable rates.

In the data analysis, we calculated the ACSH rates and the statistics describing the data variability (coefficient of variation, systematic coefficient of variation, weighted coefficient of variation and the ratio of variation) [11-13]. The Chi-square test was applied to determine if there were significant differences between observed and expected hospitalizations, and Student's t was used to test for differences in the ACSH rates by sex. The Pearson correlation was calculated to test for associations among the different ACSH. Point graphs and maps were designed to represent the ACSH rates in the different health districts.

## Results

For the years 2001–2003, the CMBD-AH registered a total of 390,017 hospital discharges for the study group of persons 65 years and older in the 34 health districts of the CM. The study population was 60% female and the mean age was 78.9 years (76.9 years in men and 80.5 years in women). Of all the hospitalizations, 16.5% (64,409) were ACSH.

**Figure 1:**
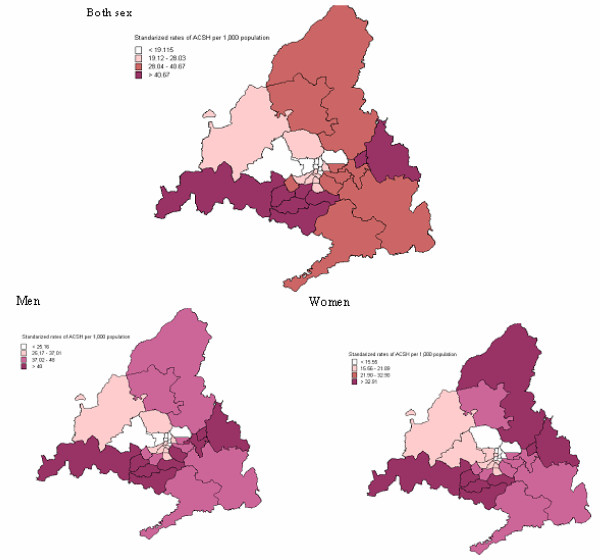
Map of standardized ACSH rates grouped by cuartiles, by health district.

Table 2 shows the ACSH rates per 1,000 population for men and women in each of the 34 health districts. The main result shown in this table is that ACSH rates were higher in men than in women, and these differences were statistically significant (p < 0.05) in each district. Men also had higher ACSH rates than women in each age group studied. These differences were studied by health district, and statistically significant differences were found in each age group (p < 0.05). Figure 1 shows the standardized rates grouped by quartiles for all ACSH in the 34 health districts. A centripetal pattern can be observed, with lower rates in the districts in the center of the CM. This geographic distribution is maintained after grouping by sex.

**Table 2:** Age-standardized* hospitalization rates for ACSCs in men and women, by health district in the Community of Madrid

	Men**		Women**	
		
Health district	Number of hospitalizations	Rates per 1,000 population	Number of hospitalizations	Rates per 1,000 population
				
101 Arganda	638	47.35	660	31.39
102 Moratalaz	1,186	43.60	976	22.01
103 Retiro	668	24.43	736	13.31
104 Vallecas	2,632	48.80	2,601	28.15
201 Coslada	492	42.47	571	32.74
202 Salamanca	710	19.66	688	8.30
203 Chamartín	565	17.66	585	9.73
301 Alcalá de Henares	1,443	60.72	1,325	38.41
302 Torrejón de Ardoz	686	75.53	592	43.22
401 Ciudad Lineal	1,380	28.08	1,250	14.88
402 San Blás	1,148	38.10	1,066	22.92
403 Hortaleza	654	23.11	628	14.00
501 Alcobendas	1,292	47.73	1,289	33.38
502 Colmenar Viejo	402	37.69	417	25.66
503 Tetuan	733	20.76	727	10.89
504 Fuencarral	909	25.40	906	15.28
601 Majadahonda	469	22.62	576	15.64
602 Collado Villalba	671	29.16	698	19.98
603 Moncloa	489	20.82	520	11.50
701 Centro	1,017	36.32	1,272	18.85
702 Chamberí	757	19.74	994	17.76
703 Latina	1,627	30.08	1,753	19.93
801 Mostoles	1,051	59.95	1,206	41.11
802 Alcorcón	871	45.44	831	26.47
803 Navalcarnero	696	54.23	751	35.71
901 Leganés	968	46.23	900	28.89
902 Fuenlabrada	883	88.48	827	51.87
1001 Parla	849	74.59	932	51.99
1002 Getafe	1,040	59.02	1,008	34.89
1101 Aranjuez	782	43.57	687	25.15
1102 Arganzuela	957	35.48	939	16.65
1103 Villaverde	868	34.91	800	21.77
1104 Carabanchel	1,822	34.95	1,724	19.72
1105 Usera	866	30.84	753	16.57

Data source: CMBD-AH, 2001–2003. Health Information Service of the General Directorate of Informatics, Communications and Technological Innovation of the Regional Health Ministry of the Community of Madrid.

ACSC: Ambulatory Care Sensitive Conditions * Rates standardized by the direct method ** p < 0.05 with Student's t-test

Table 3 shows the rates and variation statistics for all ACSH. Globally, the rate was higher among men: 33.15 per 1,000 population vs. 22.10 in women. For men the range was 70.82 and the coefficient of variation (CV) was 0.47, while for women the range was 43.69 and the CV was 0.48. In both cases the systematic coefficient of variation (SCV) was larger than 0.20.

**Table 3:** ACSH rates and variation statistics, by sex. Community of Madrid

		All ACSC hospitalizations		
		
		Both sexes	Men	Women
		
N	Health districts	34	34	34
	Population	2,428,373	972,093	1,456,280
	Cases	64,409	32,221	32,188
Rates	Crude rate	26.52	33.15	22.10
	Adjusted rate	26.27	35.57	20.45
	Minimum	12.20	17.66	8.30
	Maximum	69.03	88.48	51.99
	Percentile 5	12.64	19.16	9.37
	Percentile 25	19.12	25.16	15.55
	Percentile 50	28.03	37.01	21.89
	Percentile 75	40.67	48.00	32.90
	Percentile 95	66.56	78.77	51.90

Variation statistics	CV	0.47	0.47	0.48
	SCV	0.33	0.20	0.37
	wCV	0.61	0.62	0.61
	Chi-square	<0.001	<0.001	<0.001

Ratio of variation	RV	5.66	5.01	6.26
	RV P5-P95	5.26	4.11	5.54
	RV P25-P75	2.13	1.91	2.12

CV: coefficient of variation. SCV: systematic coefficient of variation. wCV: weighted coefficient of variation. RV: ratio of variation. ACSC: Ambulatory Care Sensitive Conditions. ACSH: Hospitalizations for Ambulatory Care Sensitive Conditions

In 93.1% of cases, the ACSH were caused by hypertensive cardiovascular disease, heart failure or pneumonia. The remaining 6.9% were divided among several causes, most notably, 3.9% for ulcer and 1.1% for diabetes mellitus. In 36.3% (23,375) of cases, the hospitalizations were for hypertensive cardiovascular disease; 56.4% of these were in men and 43.6% in women (p < 0.001). Some 35.5% of hospitalizations (22,863) were for heart failure, 37.3% of which were in men and 62.7% in women (p < 0.001). About 21.3% of all ACSH (13,749) were for pneumonia, 60.4% in men and 39.6% in women (p < 0.001).

Table 4 shows the rates and variation statistics for each of the three most frequent causes of hospitalizations. In all three cases, the lowest adjusted rates were found in women. All three types of hospitalizations showed a high level of variability. The lowest variability was seen for hypertensive cardiovascular disease (SCV = 0.23), and the highest for pneumonia (SCV = 0.69), both after adjusting for sex and when calculated separately for men and women. As can be seen, pneumonia was the condition with the widest variability in ACSH rates.

**Table 4:** ACSH rates and variation statistics, by cause of hospitalization.

		Hypertensive cardiovascular disease			Heart failure			Pneumonia		
		
		Both sexes	Men	Women	Both sexes	Men	Women	Both sexes	Men	Women
		
N	Health districts	34	34	34	34	34	34	34	34	34
	Population	2,428,373	972,093	1,456,280	2,428,373	972,093	1,456,280	2,428,373	972,093	1,456,280
Rates	Cases	23,375	13,189	10,186	22,863	8,538	14,325	13,749	8,298	5,451
	
	Crude rate	9.41	8.78	9.84	9.63	13.57	6.99	5.66	8.54	3.74
	Adjusted rate	9.30	9.66	8.97	9.58	13.94	6.65	5.59	9.57	3.39
	Minimum	3.64	7.30	2.48	3.64	3.84	2.96	1.1	1.92	0.75
	Maximum	27.39	41.09	18.42	25.38	25.21	25.65	16.96	28.71	9.81
	Percentile 5	3.74	7.94	2.95	3.74	4.61	3.45	1.27	2.04	0.86
	Percentile 25	8.37	11.71	5.67	6.79	6.63	6.66	3.23	5.38	2.14
	Percentile 50	10.02	14.60	6.86	9.50	9.82	9.75	5.27	8.80	3.54
	Percentile 75	13.59	18.36	9.65	14.39	14.23	15.47	10.17	15.26	6.78
	Percentile 95	20.38	28.44	15.15	21.36	20.97	22.14	15,94	26.39	9.58
	
Variation statistics	CV	0.43	0.44	0.46	0.51	0.55	0.50	0.61	0.62	0.61
	SCV	0.23	0.16	0.28	0.32	0.22	0.38	0.69	0.57	0.77
	wCV	0.54	0.55	0.57	0.69	0.74	0.67	0.84	0.85	0.84
	Chi-square	<0.001	<0.001	<0.001	<0.001	<0.001	<0.001	<0.001	<0.001	<0.001
	
Ratio of variation	RV	7.52	5.63	7.43	6.97	6.57	8.67	15.28	14.95	13.08
	RV P5-P95	5.45	3.58	5.14	5.72	4.55	6.42	12.58	12.94	11.10
	RV P25-P75	1.62	1.57	1.70	2.12	2.15	2.32	3.15	2.84	3.16

CV: coefficient of variation. SCV: systematic coefficient of variation. wCV: weighted coefficient of variation. RV: ratio of variation. ACSH: Hospitalizations for Ambulatory Care Sensitive Conditions

**Figure 2:**
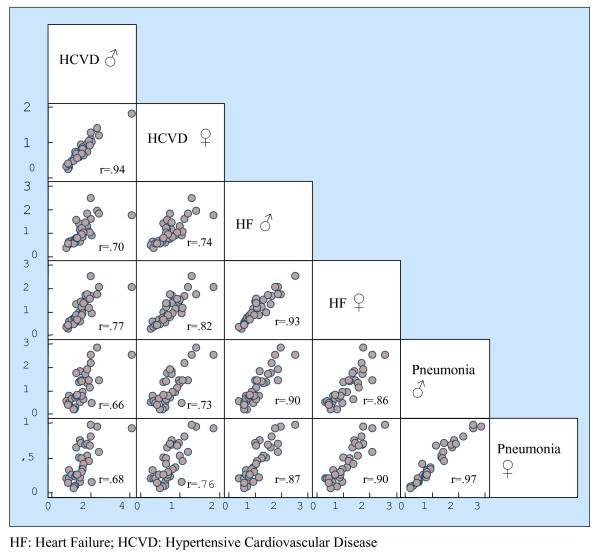
Correlation of rates of ACSC avoidable hospitalizations.

The coefficients of correlation were calculated between the different causes of hospitalization. The results were 0.91 between the hospitalization rate for heart failure and pneumonia, 0.80 between hypertensive cardiovascular disease and heart failure, and 0.73 between hypertensive cardiovascular disease and pneumonia. The correlations for these diseases by sex are shown in Figure 2.

## Discussion

This study demonstrates the existence of high ACSH rates in the elderly population, and that these rates are higher in men than in women. It also shows that there is considerable variability in these rates, even in a health system like Spain's, which offers universal coverage.

The ACSH rates found are similar to those obtained in studies in other countries for this age group [14-17], and are higher than those observed in persons under 65, both in Spain [18-20] and in other countries [21-23]. The ACSH rates in older men are higher than those in women, both globally and for the three most frequent avoidable causes of hospitalization, a finding that is consistent with the results of other studies [1,14]. Factors associated with different patterns of morbidity and use of services by men and women as well as sex-linked factors may explain this finding[24]. With regard to the differences in morbidity between men and women, men were seen to have a higher prevalence of diseases like heart failure, asthma or chronic bronchitis, which were included in the list of ambulatory care sensitive conditions (ACSC) used in this study, while women had a higher prevalence of non-fatal chronic diseases (arthrosis, osteoporosis, and osteomuscular problems and depression in general), conditions that were not included in the ACSC list but may lead to both lower quality of life and lower mortality [25-27] than in men. On the other hand, studies have shown that women use primary care services more than men do, whereas they make less use of hospital services[28,29]. The gender-associated role of family caregiver may also lead some women to reject hospitalization out of a need to meet their caregiver responsibilities[24].

However, there is a high correlation between ACSH rates in men and women by districts, that is, districts with higher numbers of admissions for men also have higher numbers of admissions for women. This may indicate the existence of a common factor such as deficiencies in PHC, different admission policies in the reference hospitals in these districts, or sociodemographic differences.

Considerable variation in ACSH rates in persons 65 years or older has also been reported by other authors[14,22,30-32], however it is less pronounced than what has been found in persons under 65[23,33-35]. Our study found less variability than that described in studies of the Medicare population[34]. These differences may be due to factors that depend on characteristics of the population, of health providers or of both [36]. The difference between our results and those of other studies could be attributed to the fact that that the global ACSC indicator used in our work is different from the list used in studies in the United States. This would be the case if we had used only the whole list with all the ACSC codes. However, the differences were maintained when we looked at the breakdown by specific conditions, such as pneumonia, in which the diagnostic codes are similar to those used in other studies [7,21,37].

Some methodological aspects of this work require a comment. First, it was based on secondary data; therefore it has limitations with regard to the validity of the principal diagnosis at discharge and the level of completeness of some of the important variables, such as home address, which limit the georeferencing of a large number of cases.

Second, the study was made only in public hospitals, which represent 71% of the hospitalizations in the CM. We chose not to include data collected in private hospitals for two main reasons: a) The data provided were frequently incomplete; b) It had previously been shown that inclusion of data from private hospitals had no impact on the ACSH rates (correlation coefficient of 0.938 between the datasets with and without private hospitals) [35].

Third, the three CMBD-AH data files did not contain all the variables needed to identify specific patients, therefore it was not possible to eliminate readmissions [11,12].

Fourth, because this study was based on aggregate data, it should be noted that an association among variables at an aggregate level does not necessarily mean that the association exists at the individual level[38].

Fifth, the indicator used has been validated for Spain[10], therefore this should not constitute a limitation. The list of ACSCs used is not specific to the elderly population, which could represent a limitation on its use in that population, mainly for chronic clinical conditions so severe that even patients with appropriate access to PHC could not have avoided hospitalization.

ACSH have been used by a variety of authors as an indirect measure to evaluate different aspects of the health system. In the United States, the ACSH indicator has been used to study and identify problems related with access to ambulatory care. Thus, areas with high rates of ACSH would have greater problems of access than those with lower rates. Various studies in the United States have found an inverse relation between ACSH rates and various indicators such as having medical insurance[37,39], income level[21,22,40], and related variables (race[41,42], residence in rural or urban areas[43,44], etc).

The National Health Service in the United Kingdom uses ACSH rates as an indicator of the quality of primary care[43,45]. It has been proposed to use this indicator in Spain, initially, for the same purpose as in previous studies that have focused on the pediatric [46,47], and general population [18,19,48]. However, this use of the indicator to evaluate the quality of the Spanish primary care model is subject to debate[19] because the results may be influenced, not only by the capacity to reduce health problems in Primary Care, but also by other variables, such as morbidity, different patterns of use of health resources, and the use of specialist care[49].

In light of the foregoing, Spanish researchers are increasingly proposing that the ACSH indicator be used to evaluate access to health services. Although the Spanish health care system provides universal coverage, in practice equal access does not exist [6] especially for older people, who are the main users of primary care. The results of this paper point in this direction.

Although other studies are needed, our data show small-area differences in ACSH rates in the CM, despite the region's relative homogeneity. It would be useful to have more precise knowledge of the present situation and to identify the factors (number of physicians, transportation time to the health center, income, educational level, differences in morbidity, disability...) that may influence the variability in avoidable hospitalizations in the elderly population. It is hoped that this study will encourage the use of this indicator as a way to detect problems associated with access to care in a health system with universal coverage.

## Conclusion

In older people in the Community of Madrid, avoidable hospitalizations for ACSC were responsible for a considerable proportion of hospital admissions occurring in the study period, amounting to about 16.5%. The most frequent causes of these hospitalizations were, in decreasing order, hypertensive cardiovascular disease, heart failure and pneumonia. Wide variability was found in ACSH rates by sex (higher in men) and by geographic areas of analysis.

## Abbreviations

ACSC, Ambulatory Care Sensitive Conditions; ACSH, Hospitalizations for Ambulatory Care Sensitive Conditions; CM, Community of Madrid; CMBD-AH, Minimum Basic Data set on Hospital Discharges and Ambulatory Surgery. SCV, Systematic Coefficient of Variation; CV, Coefficient of Variation. ICD-CM, International Classification of Diseases 9^th ^revision, Clinical Modification. PHC, Primary Health Care. RV, Ratio of Variation. wCV, Weighted Coefficient of Variation. HCVD, Hypertensive Cardiovascular Disease. HF, Heart Failure.

## Competing interests

The author(s) declare that they have no competing interest.

## Authors' contributions

PM, AA and AO conceived the study and participated in its design. JMR helped write the manuscript. PM performed the analysis and wrote the first draft. All the authors read and approved the final manuscript.

## Pre-publication history

The pre-publication history for this paper can be accessed here:


